# Nanotubes from Transition Metal Dichalcogenides: Recent Progress in the Synthesis, Characterization and Electrooptical Properties

**DOI:** 10.1002/smll.202400503

**Published:** 2024-07-02

**Authors:** Lena Yadgarov, Reshef Tenne

**Affiliations:** ^1^ The Department of Chemical Engineering Ariel University Ramat HaGolan St 65 Ariel 4077625 Israel; ^2^ Department of Molecular Chemistry and Materials Science Weizmann Institute Hertzl Street 234 Rehovot 7610010 Israel

**Keywords:** Janus nanotubes, MoS_2_, nanotubes, transition‐metal dichalcogenide compounds (TMDC), WS_2_

## Abstract

Inorganic layered compounds (2D‐materials), particularly transition metal dichalcogenide (TMDC), are the focus of intensive research in recent years. Shortly after the discovery of carbon nanotubes (CNTs) in 1991, it was hypothesized that nanostructures of 2D‐materials can also fold and seam forming, thereby nanotubes (NTs). Indeed, nanotubes (and fullerene‐like nanoparticles) of WS_2_ and subsequently from MoS_2_ were reported shortly after CNT. However, TMDC nanotubes received much less attention than CNT until recently, likely because they cannot be easily produced as single wall nanotubes with well‐defined chiral angles. Nonetheless, NTs from inorganic layered compounds have become a fertile field of research in recent years. Much progress has been achieved in the high‐temperature synthesis of TMDC nanotubes of different kinds, as well as their characterization and the study of their properties and potential applications. Their multiwall structure is found to be a blessing rather than a curse, leading to intriguing observations. This concise minireview is dedicated to the recent progress in the research of TMDC nanotubes. After reviewing the progress in their synthesis and structural characterization, their contributions to the research fields of energy conversion and storage, polymer nanocomposites, andunique optoelectronic devices are being reviewed. These studies suggest numerous potential applications for TMDC nanotubes in various technologies, which are briefly discussed.

## Graphical abstract

This review describes recent (mostly last five years) progress in the synthesis, structural characterization, and properties of nanotubes from layered transition metal dichalcogenides, including in‐silico investigations. In the picture, ultra long multiwall nanotubes (up to 0.5 mm) nanotubes reported in Ref. 27 are shown via SEM (bottom) TEM (right) and electron diffraction (top).

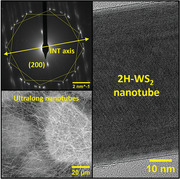

## 1 Introduction

Following the discovery of carbon nanotubes by Iijima, multiwall nanotubes from the inorganic compound WS_2_, which possesses a layered structure, were reported shortly afterward.^[^ ^1^ ^]^ This early study was followed by a series of other works,^[^ ^2^, ^3^, ^4^, ^5^, ^6^, ^7^, ^8^, ^9^ ^]^ propelling the idea that nanoparticles (NPs) of inorganic compounds with layered structures (2D materials) are inherently unstable in the flat morphology. It is well accepted by now that nanoparticles of layered compounds spontaneously fold and seam under proper conditions, forming two generic structures, i.e., quasi‐spherical (or polyhedral) nanostructures denoted inorganic fullerene‐like (IF) and nanotubes (NTs or INT). The large strain involved in folding the triatomic S‐W(Mo)‐S layer was shown to be compensated by expanding the radius of the nanotubes (>10 nm) compared with CNT and the preferred multiwall structure.^[^ ^10^ ^]^ Synthesis of inorganic nanotubes from 2D materials generally relies on high‐temperature chemical reactions, which are case‐specific and challenging to control. Therefore, only in a few cases sufficient understanding of the growth conditions permitted the production of pure phases of such nanotubes. Fortunately, the synthesis of multiwall WS_2_ (and also WSe_2_) nanotubes went a long way since they were first reported.^[^ ^11^, ^12^ ^]^ This progress allowed the fabrication of copious amounts of pure phases of such nanotubes using a lab‐size CVD (horizontal) flow reactor or a fluidized bed (vertical) reactor, within a day's work. With some clues remaining unsolved, the mechanism of the WS_2_ nanotubes synthesis from WO_3‐x_ nanoparticles and particularly from W_18_O_49_ nanowhiskers was established,^[^ ^11^, ^12^ ^]^ enabling scaling‐up of its production.^[^ ^13^ ^]^ Equally important, the synthesis of BN nanotubes (NTs) also made great strides forward with the production of a few tens of gh^−1^ using ammonia borane and a hot plasma‐assisted reactor.^[^ ^14^ ^]^ In rudimentary terms, the sulfidation reaction starts from the surface of the tungsten oxide nanoparticle/nanowhiskers, progressing inwards in layer‐by‐layer mode according to a “surface‐inwards” mechanism.^[^ ^15^, ^16^ ^]^ While the first two or three tungsten sulfide layers are formed within a few seconds at 840 °C, the deeper the reaction goes into the oxide core, the slower it becomes, necessitating a few hours of annealing period or alternatively using higher annealing temperatures. Here, the oxide nanoparticle/nanowhisker serves as a self‐sacrificing template onto which the closed sulfide layers of the nanotubes (and IF) are formed.

Nonetheless, limited control over the diameter, number of layers, and chiral angle of the WS_2_ nanotubes has been achieved so far. While for most studies, singlewall nanotubes with well‐defined chiral angles are desired, other works benefited from the structural polydispersity of these multiwall nanotubes. The following perspective intends to review the recent progress in the research of transition metal dichalcogenide (TMDC) nanotubes and that of WS_2_ in particular. A few aspects related to the advancements in the synthesis of nanotubes from pure binary TMDC compounds with layered structure and ternary alloys thereof will be described. Recent studies using such nanotubes shed light on new optical and electrooptical characteristics of such nanotubes, offering hitherto unexplored potential applications. These, together with some mechanical aspects and structural characterization of metal dichalcogenide nanotubes, are the premier goals of this short review.

## 2 Advancements in the Synthesis

**Figure 1:**
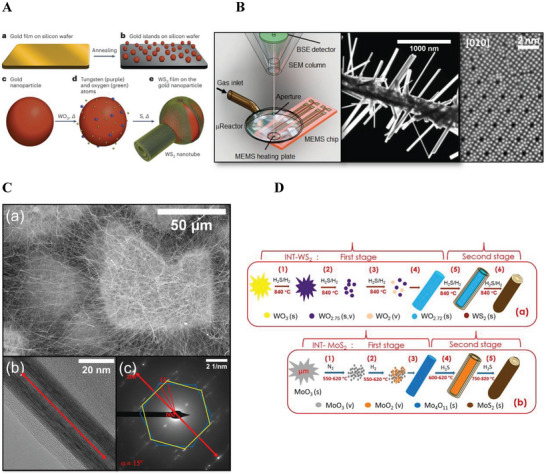
A: a) Gold film deposited on a silicon wafer. b) Annealing and formation of gold nanoparticles. c) Schematic rendering of a gold nanoparticle. d) Deposition of a thin WO_3–_ *_x_* film onto the gold nanoparticle. e) Sulfidation and growth of the achiral multiwalled WS_2_ nanotube. *Δ*, heating. Reproduced with permission from Ref. [17, 18] Copyright 2023, Springer Nature Ltd.; B: a) Scheme of the µReactor in the SEM. b) SEM image of the γ‐WO_3_/*a*‐SiO_2_ nanofibers heat treated in the µReactor within the SEM (maximum temperature 800 °C, 100 Pa of H_2_). Figure adapted with permission from Ref. [23] Copyright 2024, ACS Publications; C: Electron microscopy analysis of WS_2_ nanotubes produced by sulfidation of the W_5_O_14_ nanowhiskers. a) SEM image of ultralong WS_2_ nanotubes. b) Transmission electron microscope (TEM) image of the WS_2_ nanotube with a marked axis along the cavity direction. The interlayer distance was measured as 0.625 nm. c) corresponding SAED measurement typical for the hexagonal structure of the WS_2_ nanotube in chiral (blue hexagon, ≈15°) and armchair conformation (yellow hexagon) of the WS_2_ layers. The figure adapted with permission from Ref. [24] Copyright 2023, ACS Publications; D: a) Schematic representation of the multistep growth mechanism of WS_2_ nanotubes^[^ ^25^ ^]^ according to the following sequence: WO_3_ → WO_2.75_ → WO_2_ + WO_2.75_ → WO_2.72_ → WS_2_. All the reaction steps are carried out at constant (840 °C) temperature and gas flow conditions. b) Schematic illustration of the proposed growth mechanism for MoS_2_ nanotubes. Different steps of this reaction are carried out at different reaction parameters: steps (1)–(3), at *T* = 550–620 °C under N_2_/H_2_ flow; step (4), at *T* = 600–620 °C; and step (5), at 750–820 °C under N_2_/H_2_S flow; N_2_ is used as a carrier gas. Evolution of molybdenum oxide into MoS_2_ nanotube in this reaction follows the sequence MoO_3_ → MoO_2_ + MoO_3_ → Mo_4_O_11_ → MoO_2_ + MoS_2_ → MoS_2_; Designations: (v) vapor, (s) solid. The figure was adapted with permission from Ref. [26] Copyright 2020, ACS Publications.

A few new approaches have been recently designed to synthesize multiwall WS_2_ nanotubes (MWNT or MWINT). Most strikingly, in a recent study, MWNT WS_2_ nanotubes, largely achiral (zigzag or armchair), have been obtained using a classical chemical vapor deposition (CVD) reactor and vapor‐liquid‐solid (VLS) growth mode.^[^ ^17^, ^18^ ^]^ In this work (schematically illustrated in **Figure** **1**A), gold nanoparticles were deposited first on a Si wafer (Figure 1A(a,b)). Then, heated WO_3_ vapors were deposited on the Si substrate, coating the gold nanoparticles with ultra‐thin (≈4 nm) film (Figure 1A(c,d)). Subsequently, sulfur vapors and hydrogen gas were allowed to effuse into the CVD reactor, while maintaining the supply of tungsten oxide vapors. The limited solubility of tungsten in gold and its catalytic reactivity with respect to sulfur led to a surface reaction converting the WO_3_ into WS_2_, which increasingly protruded from the gold nanoparticles, forming MWNT with largely achiral angle (Figure 1Ae). This facile growth technique of MWNT paves the way for the growth of achiral WS_2_ nanotubes in preselected positions, which can be beneficial for field emission sources or polaritonic waveguides, or for photocatalysis, to state a few examples.^[^ ^18^ ^]^ Moreover, in principle, this VLS‐based process can be exploited for the growth of chalcogenide nanotubes from refractory transition metals like Hf and Ta, which oxides do not exhibit sufficiently high vapor pressure at the relevant temperatures (800–1200 °C). To that end, volatile metal halides could be the starting point for such synthesis. Finally, such achiral nanotubes can be modeled more easily than nanotubes with polydispersed chiral angels, which is extremely important for comparing experimental and theoretical results. Generally speaking, the huge unit cell of multiwall inorganic nanotubes with variable chiralities is prohibitive for density functional theory (DFT)‐based calculations. Therefore, having multiwall WS_2_ nanotubes with unique chiral angle is a boon for in‐silico studies. Multiwall WS_2_ nanotubes can be synthesized directly via a one‐pot reaction of WO_3‐x_ nanoparticles with H_2_S.^[^ ^11^, ^12^ ^]^ Alternatively, they can be produced in a two‐step process in which W_18_O_49_ nanowhiskers are grown first and subsequently sulfurized and converted into nanotubes.^[^ ^19^ ^]^ The great advantage of this two‐step process is that it gives extra flexibility to tune the diameter of the nanowhiskers first,^[^ ^20^ ^]^ thereby gaining nanotubes with small and uniform diameters.^[^ ^21^ ^]^ While WS_2_ nanotubes with a diameter <15 nm were recently reported by such a two‐step process, in this specific work,^[^ ^21^ ^]^ highly crystalline WS_2_@MoS_2_ core‐shell nanotubes with a diameter <20 nm were obtained. WS_2_ nanotubes were also synthesized with sulfur vapor and under low‐pressure argon gas flow at particularly low temperatures of 650 °C.^[^ ^22^ ^]^ This temperature is particularly low compared to the ubiquitous synthesis of multiwall WS_2_ nanotubes at temperatures of 840 °C and above.

The ability to grow WO_3‐x_ in‐situ within scanning electron microscope (SEM) and transmission electron microscope (TEM) under “realistic” conditions has received some attention with the development of a new micro reactor named the µReactor (Figure 1B).^[^ ^23^ ^]^ More recently, high‐temperature in‐situ sulfidation of such nanowhiskers into multiwall WS_2_ nanotubes was demonstrated in the SEM.^[^ ^27^ ^]^ Moreover, the µReactor permits transferring the sample back and forth between the SEM and the TEM, allowing detailed structural analysis while the reaction is progressing. Given the high reactivity of heated H_2_S toward metallic parts, the µReactor was carefully designed to prevent any leak of such and future toxic gases during the synthesis. Obviously, the technology of the µReactor will enable studying the growth of different nanostructures at elevated temperatures, in situ and ex situ, by TEM permitting in‐depth evaluation of their growth mechanism.

In another recent development, WS_2_ nanotubes a few hundred micrometers long (up to 0.5 mm) and aspect ratio > 2000 were obtained in high yields (see Figure 1C) using a two‐step synthesis.^[^ ^24^ ^]^ Here, long and slim (mostly 40 nm) W_5_O_14_ nanowhiskers were obtained first by reacting the WO_2.92_ powder in a closed ampoule and under high pressure (≈4 bars) at 800 °C. Subsequently, the few hundreds of microns long tungsten oxide nanowhiskers were annealed in the flow reactor under H_2_S gas flow, which converted the entire powder of the W_5_O_14_ nanowhiskers into ultra‐long nanotubes. The crystalline perfection of the nanotubes can be appreciated from the TEM image **(b)**. The electron diffraction (SAED) of these nanotubes **(c)** shows that, while some of the walls in this nanotube are achiral (yellow) with the *a*‐axis coinciding with the axial direction (armchair configuration), other walls (cyan) exhibit chiral orientation. A web was prepared from the long interwoven nanotubes and was used to fabricate “buckypaper,” which was mechanically stable enough to self‐support itself and serve as a membrane or filter. This buckypaper was used to effectively filtrate gold NPs (≈4 nm) from aqueous solution. It is likely that other heavy metal ions/clusters could also be filtered using this buckypaper, owing to the chemical affinity of heavy metals toward sulfur.

Despite the similarity, the synthesis of WSe_2_ nanotubes received much less attention than those of WS_2_ nanotubes. Early reports^[^ ^28^ ^]^ were not followed until quite recently.^[^ ^29^, ^30^ ^]^ Here, W_18_O_49_ nanowhiskers were prepared first and selenized at elevated temperatures. The reaction with the tungsten oxide nanowhiskers proceeded from the surface inwards. Since the selenium atom is larger than the sulfur atom and less ionic, its diffusion into the inner oxide core is slower. Hence, only thin diameter (20 nm) tubes can be prepared according to this procedure. Nanotubes from the ternary alloy WS_2(1‐_ *_x_*_)_Se_2_ *_x_* (denoted as WSSe for simplicity) with 0 ≤ x ≤ 1 and random sulfur and selenium distribution in the lattice were recently studied.^[^ ^31^, ^32^ ^]^ These nanotubes were prepared by co‐sulfidation‐selenization of W_18_O_49_ nanowhiskers. The nanotubes exhibited tunable bandgap, which went down (mostly) monotonically from 2.0 eV for pure WS_2_ to 1.6 eV for the pure WSe_2_ nanotubes.

MoS_2_ nanotubes were synthesized early on using the chemical vapor transport (CVT) technique.^[^ ^2^ ^]^ Here, iodine served as the growth promoter with the source material (MoS_2_ powder) placed at a high temperature (1000 °C) at the end of the ampoule. The nanotubes grew at the lower temperature zone (800 °C), with the iodine shuttling back and forth between the hot and colder zones. Despite the progress with the synthesis of WS_2_ (WSe_2_) nanotubes from tungsten oxide nanowhiskers and the hypothetical similarity between the two, the synthesis of MoS_2_ nanotubes from molybdenum oxide nanoparticles/nanowhiskers was not well understood remaining enigmatic for many years. This puzzle was recently resolved in an exemplary methodological investigation, permitting reproducible synthesis of pure multiwall MoS_2_ nanotube phases.^[^ ^26^ ^]^ In stark contrast with the constant temperature‐gas flow regime used for the conversion of W_18_O_48_ nanowhiskers into multiwall WS_2_ nanotubes, here, the temperature was gradually raised from 550 to 820 °C. The gas flow was also varied sequentially from N_2_ to H_2_ and finally to H_2_S during the conversion of the Mo_4_O_11_ nanowhiskers into multiwall MoS_2_ nanotubes (see Figure 1D). An alternative strategy for synthesizing MoS_2_ nanotubes with a diameter >110 nm was presented in Ref. [33] Here, SiO_2_ nanowires, obtained via the electrospinning process, served as a template and were sheathed conformably by a thin concentric MoS_2_ layer using a CVD process. Subsequently, SiO_2_@MoS_2_ core‐shell 1D nanostructures were fabricated via high‐temperature sulfidation of the molybdenum oxide film. Finally, the SiO_2_ nanowire templates were removed by etching in HF solution, leaving behind hollow MoS_2_ nanotubes.

The synthesis of nanotubes consisting of single, or a few layers has seen some advances in recent times. However, the yield of such reactions is far too small to permit systematic studies of the nanotubes' properties. Fundamentally, such nanotubes (and IF nanoparticles) can be produced at ultra‐high temperatures or in far‐from‐equilibrium conditions under highly exergonic environment, such as pulsed laser ablation,^[^ ^34^ ^]^ arc discharge in aqueous solutions,^[^ ^35^ ^]^ plasma treatment of multiwall WS_2_ nanotubes,^[^ ^36^ ^]^ and focused solar ablation.^[^ ^37^ ^]^ The hot atomic soup formed under these highly exergonic conditions cools down rapidly, stumbling into windows of relative stability, characteristic of such metastable nanostructures. Further relaxation leads to the “freezing‐out” of such metastable nanostructures in ambient conditions. In this context, recent progress has been recorded in synthesizing single to triple‐layer MoS_2_ nanotubes using the high‐temperature reaction between MoO_3_ and MoS_2_.^[^ ^38^ ^]^ The meager yields did not permit more than rudimentary TEM analyses, though. An alternative approach for synthesizing MoS_2_ nanotubes using a turbulent flow reactor was recently reported.^[^ ^39^ ^]^ A turbulent flow regime has been used in the past for the growth of WS_2_ in the (vertical) fluidized bed reactor,^[^ ^13^ ^]^ which promotes frequent encounters between the reactive molecules at the nucleation sites.

Fabrication of core‐shell nanotubes using different synthetic techniques has been reported in the past.^[^ ^40^, ^41^, ^42^, ^43^, ^44^, ^45^, ^46^ ^]^ More recently, however, significant progress was recorded in synthesizing various core‐shell nanotubes. Most remarkably, Maruyama and co‐workers have fabricated three‐shell SWCNT@BNNT@MoS_2_ nanotubes with 3–5 nm diameters.^[^ ^47^ ^]^ Singlewall carbon nanotubes (SWCNT) 1–1.2 nm in diameter served as the basic template for the growth. A concentric boron nitride shell consisting of one to eight BN layers was grown on top of the SWCNT using a chemical vapor deposition (CVD) reaction between boric acid and ammonia at elevated temperatures. The thickness of the BN concentric shell was adjusted via the CVD growth time. Finally, a top single‐layer MoS_2_ nanotube was obtained by standard CVD method using MoO_3_ and sulfur vapors as precursors (**Table** **1**). Table 1 summarizes some of the main routes for the synthesis of pure molybdenum‐ and tungsten‐disulfide nanotubes, their alloys and core‐shell nanotubular structures.

**Table 1:** A summary of the main routes for the synthesis of TMDC nanotubes.

	Precursors	Temperature range (°C)	Diameter range in nm (Average)	Refs	Comments and typical amounts
WS_2_	1. Obtaining W_20_O_58_ NPs (W_18_O_49_ nanowhiskers); 2. Sulfidation by H_2_S in reducing conditions	840	30–100	[11]	Horizontal flow reactor ≈10 mg
MS_2_ (M = W,Mo)	Decomposition of MS_3_	900–1100	30–100	[6]	Flow reactor Few mg
MS_2_ (M = Ta, Nb)	Decomposition of MS_3_	700–850	30–100	[6]	Evacuated quartz ampoule Few mg
WS_2_	Reacting WO_2.7_ nanowhiskers and H_2_S	1100	30–100	[7]	Open and closed tips
MoS_2_	Chemical vapor transport with iodine	1000 and 800	Family 1 > 1 mm Family 2 ≈100 nm	[9]	3R polytype 2H polytype
MWCNT@WS_2_	Annealing WO_3_ coated MWCNT in H_2_S	900	20	[12]	Single and double wall WS_2_ nanotubes in a few mg
WS_2_	Sulfidation of WO_2.83_ NPs with H_2_S	840	30–150	[13, 25]	Vertical Fluidized bed reactor (100 g of pure NTs)
WS_2_, WSe_2_	WO_3‐x_ evaporation and then sulfidation on gold NPs‐coated Si substrate	Temperature gradient‐ Si substrate at 835	30–100	[17]	Almost 80% achiral (3R polytype)
WS_2_@MoS_2_ core‐shell nanotubes	1. W_18_O_49_ nanowhiskers prepared (880 °C); 2. WS_2_ nanotubes at 500–700 °C; 3. Thin MoO_3_ layer sputtering; 4. Sulfurization at 600–700 °C.	Multi‐step process 500–900	<20 nm	[21]	Tiny amounts (≈1 mg) of highly crystalline core‐shell nanotubes
Ultralong WS_2_ nanotubes	1. High pressure synthesis of ultralong W_5_O_14_ nanowhisker (800 °C) in quartz ampoule; 2. Their sulfidation at 845 °C in a flow reactor	Two‐step 800 850	20‐40 nm and up to 0.5 mm long	[24]	50 mg
MoS_2_ nanotubes	Multi‐step one‐pot process; temperature and gas composition are varied continuously	550–820	(60 nm)	[26]	50 mg
WS_2(1‐x)_Se_2x_	Sulfidation‐selenization of W_18_O_49_ nanowhiskers In either a tube furnace or quartz ampoule	450 to 840 ramp	(100 nm)	[31, 32]	Few tens mg
SWCNT@BN@MoS_2_ nanotubes	1. SWCNT prepared by reacting CO+ethanol at 1000–1200 °C with ferrocene as catalyst; 2. H_3_N_B_H_3_ vapors reacted at 1000–1100 °C; 3. MoO_3_+S reaction at 500–700 °C	Each process is carried out at a specific temperature	<5 nm	[47]	Few mg

SWCNT‐ Singlewall carbon nanotubes; MWCNT‐ Multiwall carbon nanotubes.

## 3 The Growth Mechanism of WS_2_ Nanotubes from Tungsten Oxide Nanoparticles

Multiwall WS_2_ nanotubes are the most studied inorganic nanotubes from layered compounds. They are available in substantial amounts and a pure phase. The most prominent pathway for their synthesis is via sulfidation of WO_3‐x_ nanoparticles through the “surface‐inwards” mechanism.^[^ ^13^ ^]^ This mechanism is schematically depicted in Figure 1D. The synthesis of MoS_2_ nanotubes follows a similar mechanism, albeit with some key differences; see Figure 1D.^[^ ^26^ ^]^ Recent advancements in in situ electron microscopy permitted gaining deeper insight into the growth mechanism of WS_2_ nanotubes through a complementary mechanism denoted as “receding oxide core.”^[^ ^27^ ^]^

The roots of the “surface‐inwards” mechanism lie in the early works of Feldman et al.^[^ ^15^, ^16^ ^]^ who described the synthesis of fullerene‐like WS_2_ (IF‐WS_2_) nanoparticles by sulfidation of WO_3‐x_ nanoparticles in a reducing atmosphere. TEM analysis of individual nanoparticles during the reaction (Figure 1 in Ref. [16]) provided unequivocal support for this mechanism. Here, following fast hydrogen diffusion inwards, the core becomes partially reduced in a matter of a few seconds. Subsequently, 2–4 closed WS_2_ layers grow quickly (1–2 min), enfolding the oxide core and passivating it so that each nanoparticle becomes, in fact, a microreactor of its own. In the third stage of the reaction, sulfur vapors diffuse inwards slowly (30–60 min) through defects in the passivating WS_2_ top layers, converting the reduced oxide core into a hollow IF‐WS_2_ nanoparticle. This advance permitted further‐on scaling‐up of the IF‐WS_2_ NPs production via the one‐pot fluidized bed reactor (FBR) and their commercialization as superior solid lubricants. Without going into too much detail, the synthesis of pure IF‐MoS_2_ was also clarified.^[^ ^48^ ^]^ It was found that owing to the volatility of MoO_3_ at the relevant temperatures (>800 °C), the synthesis of IF‐MoS_2_ NPs is appreciably more challenging to control, but in fact, the same mechanism, i.e., “surface‐inwards” for converting the reduced MoO_3‐x_ into hollow IF‐MoS_2_ NPs, holds here as well.

The growth of WS_2_ nanotubes in a two‐step process was conceived immediately afterward,^[^ ^7^, ^11^, ^12^ ^]^ laying down the foundations for their one‐pot scaled‐up synthesis at elevated temperatures (>800 °C).^[^ ^13^, ^25^ ^]^ Here, a slight reduction of the WO_2.82_ nanoparticles leads to the volatilization of tungsten oxide clusters at elevated temperatures and the fast (few tens of seconds) growth of W_18_O_49_ nanowhiskers, of typically 25–150 nm in diameter and a few tens of μm long (aspect ratio of >100). In the second step these nanowhiskers are sulfided by exposing them to a mixture of H_2_S under a reducing atmosphere. The sulfidation reaction starts at the surface of the W_18_O_49_ nanowhisker, proceeding inwards (WS_2_) layer‐by‐layer in a quasi‐epitaxial mode into the core until a hollow nanotube is obtained according to the “surface inwards” mechanism. While the first 2–4 layers form within a matter of a few minutes, the progress of the sulfidation reaction becomes very slow. Surprisingly, it was found that under specific conditions, this reaction can be made one‐pot, whereby the WO_3‐x_ NPs are converted into W_18_O_49_ nanowhiskers in a matter of a few seconds. Subsequently, the sulfidation reaction of the nanowhiskers, according to the “surface inwards” mechanism, follows, leading to hollow WS_2_ nanotubes.^[^ ^13^, ^25^ ^]^ This progress permitted mass production of such multiwall nanotubes using the fluidized bed reactor.

**Figure 2:**
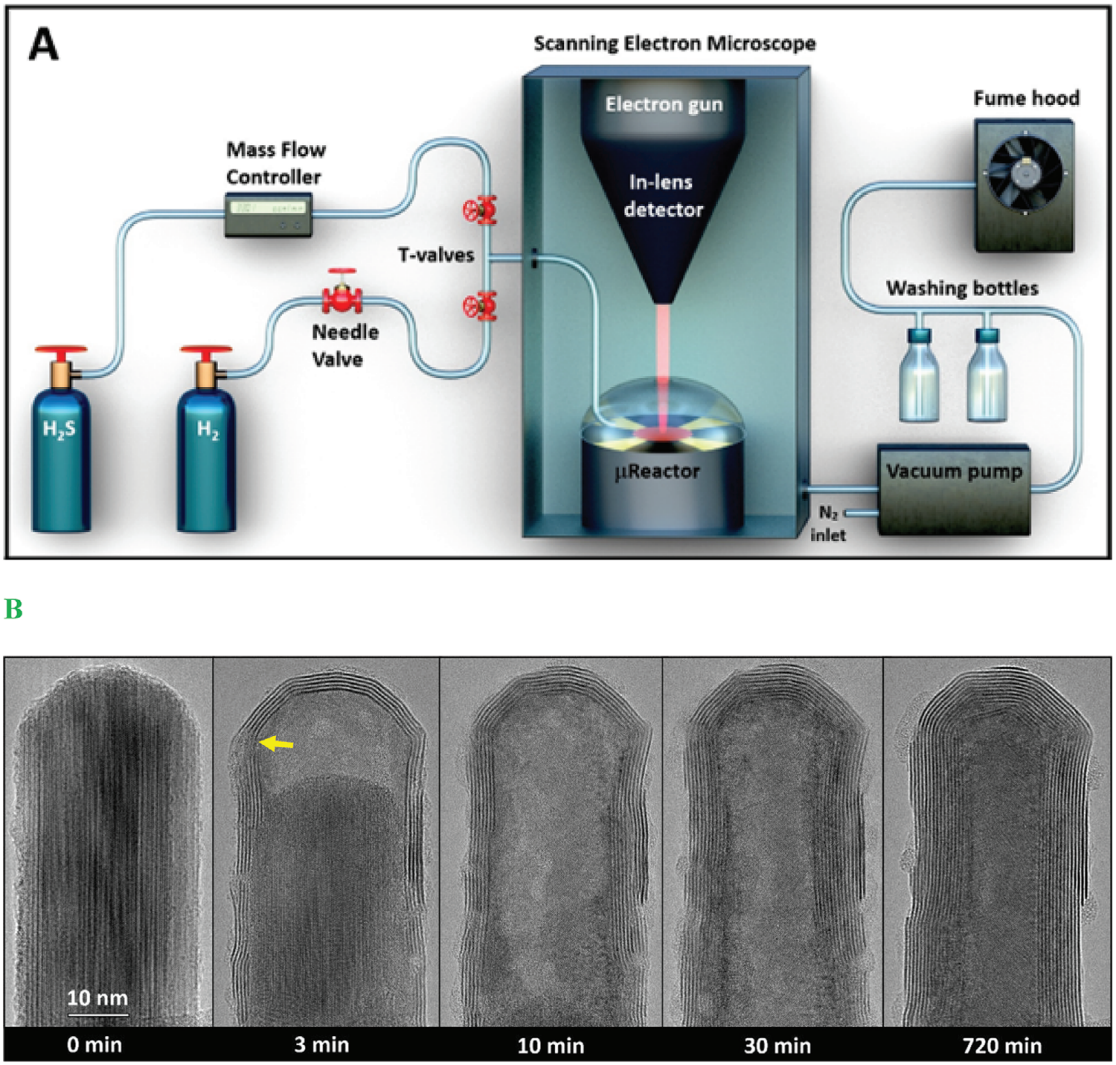
A. Schematic rendering A) of the modified SEM with a µReactor dedicated to studying sulfidation reactions at elevated temperatures. The utilized gases are hydrogen sulfide and hydrogen, which are fed through the piping and valves system. A mass‐flow controller and needle valve control the H_2_S and H_2_ supplies, respectively. The µReactor consists of a reactor body, a heating chip with a sample, and a lid for maintaining the gas pressure. The canopy of the reactor has a gas inlet and an aperture at the top for the incident electron beam and for collecting the backscattered electrons (BSE). The sulfidation reaction is inspected in situ via an in‐lens BSE detector T1. Notably, the µReactor is pressurized up to 500 Pa (5 mbar) while a system of vacuum pumps keeps the SEM chamber under a high vacuum. The final gas outlet is directed to washing bottles with sodium hydroxide solution for H_2_S scrubbing and subsequently to the fume hood. B. ex situ TEM images sequence of the sulfidation of W_18_O_49_ nanowhisker at 1000 °C in H_2_S/H_2_ (50 + 25 Pa). The pristine W_18_O_49_ nanowhisker (before the reaction) is shown (0 min). The reaction in the µReactor (within the SEM) was interrupted at preselected times (3, 10, 30, and 720 min) for the ex situ TEM analysis. The yellow arrow after 3 min reaction indicates the position of a significant defect in the WS_2_ wall. Reproduced with permission or Ref. [27] Copyright 2024, ACS Publications.

In a recent work, WS_2_ nanotubes were synthesized in‐situ in the SEM using a modified µReactor (see **Figure** **2**A), revealing what appears to be a complementary growth mechanism.^[^ ^23^ ^]^ The chip with the SiN membrane carrying the reacted sample can be switched alternately between the SEM, where the high‐temperature sulfidation of the W_18_O_49_ nanowhiskers takes place, and the TEM, permitting high‐resolution analysis of the formed nanotubes. Here, following the formation of the first 3–4 layers of WS_2_ on the surface of the W_18_O_49_ nanowhisker (according to the “surface inwards” mechanism), the tungsten‐oxide at the core starts to evaporate near the tip, forming a cavity that grows with time (the so‐called “receding oxide mechanism”). While some of the oxide vapors leak out of the nascent nanotube through defects in the top WS_2_ layers near the tip (see yellow arrow after 3 min), the remaining oxide vapor reacts with H_2_S gas, which diffuses into the cavity from outside into the core through the same defects. This reaction yields WS_2_ layers, which are deposited within the core, progressing slowly in the cavity away from the tip toward the center of the hollow core. Figure 2B displays a series of *ex‐situ* TEM images of a specific oxide nanowhisker converted gradually into a hollow WS_2_ nanotube by reacting in the in situ SEM. The “receding oxide mechanism” is clearly visible in this series of images taken at different sulfidation times from the same nanowhisker. The “receding oxide core” mechanism is particularly important at temperatures exceeding 900 °C, where the vapor pressure of tungsten oxide is very high. Further work is required to elucidate the growth mechanism of WSe_2_ and MoSe_2_ nanotubes. Admittedly, though, notwithstanding the continued progress in synthesizing WS_2_ and related nanotubes, control of their diameter, number of layers, and particularly the chirality of the walls has been limited so far. Notwithstanding these limitations, numerous applications have been offered recently for such multiwall nanotubes in various technologies.

## 4 *Ab‐Initio* Calculations of Nanotubes: Forward

Early *ab‐initio* calculations of singlewall BN^[^ ^3^ ^]^ and doublewall GaSe^[^ ^49^ ^]^ nanotubes provided the first hints on the unique structure and properties of nanotubular structures. In particular, the calculations showed that these nanotubes are semiconducting. Surprisingly, however, owing to the large effect of strain, the bandgap of these nanotubes shrinks upon reducing the nanotube diameter, which overcomes the bandgap expantion due to quantum‐size effect. These pioneering works were complemented by the early theoretical works of Seifert and co‐workers on MoS_2_^[^ ^50^ ^]^ (WS_2_) nanotubes. For the first time, evidence of a direct bandgap transition in singlewall zigzag nanotubes was provided, which was later transcended to a single‐layer MoS_2_ sheet. Also, the reduction of the (indirect and direct) bandgap with shrinking tube diameter was studied systematically. In recent years, much attention has been paid to the calculations of inorganic nanotubes of ever greater complexity_._ Significantly, the synthesis of many of the calculated nanotubes was found to be elusive, but nonetheless, these contributions provided both incentives and guidelines for their experimental study. For example, nanotubes belonging to the family of compounds MX_2_ with M = Ni, Pd, or Pt; X = S, Se, Te were investigated using DFT calculations.^[^ ^51^ ^]^ The nanotubes were found to go through an indirect to quasi‐direct transition under strain. Unfortunately, these nanotubes have not been studied experimentally so far.

## 5 Janus Nanotubes

### 5.1 Ab Initio Calculations of Janus Nanotubes

**Figure 3:**
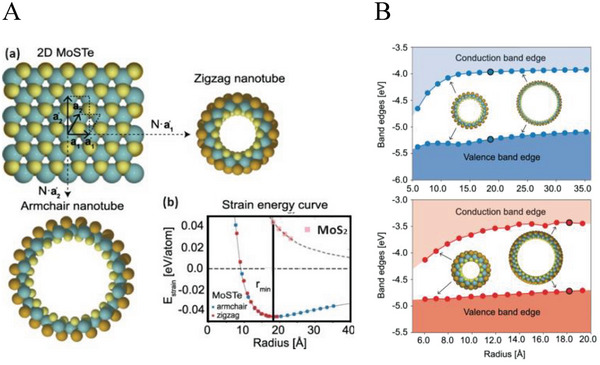
A: a). Schematic drawing of a MoSTe Janus nanosheet and its folding along the a'_1_ axis to obtain zigzag and the a'_2_ axis to obtain armchair nanotubes. b). The strain energy of such nanotubes displays a minimum around a radius of 20 Å. On the other hand, the strain energy of a MoS_2_ (MoTe_2_) nanotube is positive at any radius (adopted with changes from Figure 1 of Ref. [56]). Sketch of how armchair and zigzag nanotubes are constructed by rolling up a 2D MoSTe monolayer along different directions. b) Calculated strain energies of the armchair and zigzag MoSTe nanotubes for different radii. Minimum strain energy is attained at r_min_, which is the most stable nanotube radius. For comparison, the strain energy of a (metastable) MoS_2_ nanotube is shown. B: The position of the valence and conduction bands as a function of the tube diameter for (up) armchair and (bottom) zigzag MoSTe nanotubes. Adopted with minor changes from Figure 3 of Ref. [56] with permission from Ref. [56] Copyright 2021, Physical Review Materials, APS.

The interest in asymmetric layered compounds, like Se‐Mo‐S, known as Janus, has attracted significant attention in recent years.^[^ ^52^ ^]^ Several kinds of Janus nanotubes from TMDC compounds have been discussed in the literature in recent years; however, they are exclusively addressed through *ab‐initio* calculations.^[^ ^53^, ^54^, ^55^, ^56^, ^57^ ^]^ Here, the strain provoked by the asymmetry between, e.g., the outer selenium and the inner sulfur layers induces the spontaneous folding of the layers and their seaming into nanotubes. The optimal radius of the (singlewall) tube was determined mostly through the size difference between the nanotube's outer and inner chalcogen atoms.^[^ ^55^, ^56^ ^]^ Not surprisingly, therefore, the smallest diameter Janus nanotubes are those with sulfur in the inner (concave) layer and tellurium in the outer (convex) one, i.e., Te‐M‐S (M = Mo, W, Nb, Ta). **Figure** **3**A(a) shows a schematic drawing of a MoSTe nanosheet and nanotubes in zigzag and armchair configurations.^[^ ^55^, ^56^ ^]^ The strain energy of such nanotubes displays a minimum around a radius of 20 Å (see Figure 3A(b)), indicating the global stability of such tubes. In contrast, the strain energy of a MoS_2_ (MoTe_2_) nanotube is positive at any radius, indicating that it is a metastable structure. Indeed, MoS_2_ nanotube is more stable than a nanoribbon with the same number of atoms in a given range of radii (between, say, 20–500 Å) but is less stable than an infinitely large layer.

Janus nanotubes of the four‐center 2D materials Te‐Ga‐Ga‐S, etc., were also studied via first principle calculations.^[^ ^57^ ^]^ While the nanotubes exhibit a global (negative energy) with respect to an infinite monolayer of the same kind, it is metastable (positive energy) relative to the bulk crystal.

Figure 3B shows the evolution of the valence and conduction bands of MoSTe nanotubes with armchair and zigzag configuration.^[^ ^56^ ^]^ One notices that both bands shrink with a decreasing diameter but not uniformly. The band shrinkage can be attributed to the increased curvature (1/R) upon reducing the tube diameter (2R). Hence, the Mo‐X bonds in the nanotube get distorted, thereby reducing the overall hybridization energy leading to a shrinkage of the gap.

### 5.2 Janus Nanoscrolls

Unfortunately, the synthesis of Janus nanotubes has been elusive so far. One major reason is the difficulty in obtaining singlewall TMDC nanotubes, which can be a good starting point for the chalcogen atom exchange. Additionally, the high temperature required for such syntheses induces large entropic effects, which are maximized upon the random distribution of the chalcogen atoms in the anion lattice sites. Nonetheless, nanoscrolls from S‐Mo‐Se and S‐W‐Se were recently prepared and studied to some extent iRef. [58] First, MoSe_2_ and WSe_2_ monolayers were deposited using CVD. H_2_ plasma treatment of the layers led to the removal of the top selenium layer, which was replaced by a sulfur layer using H_2_S annealing without breaking the vacuum. Subsequently, the Janus layer was treated with dimethyl furan (DMF), which led to its separation from the Si/SiO_2_ substrate and spontaneous rolling into a nanoscroll.

In another recent study, the fabrication of narrow nanoscrolls by utilizing Janus TMDC monolayers was described.^[^ ^59^ ^]^ Generally, the Janus WSSe and MoSSe monolayers were prepared through the plasma‐assisted surface atom substitution of WSe_2_ and MoSe_2_ monolayers, respectively, and then were rolled by solution treatment.^[^ ^59^ ^]^ Atomic resolution elemental analysis confirmed that the Janus monolayers were rolled up with the Se‐side surface on the outside. The smallest diameter of the nanoscrolls was ≈5 nm, almost the same as the value predicted by the DFT calculation.

Interestingly, the scroll direction was determined by the angle with respect to the edge of the triangular grain of the TMDC. Under the prevailing growth conditions, the grain edges were aligned with the metal‐terminated zigzag edges.^[^ ^59^ ^]^ The findings of this work indicate that the crystal orientation of the nanoscrolls, corresponding to the chirality of the nanoscrolls, was essentially random. The formation of the nanoscrolls was found to be significantly influenced by the number of layers in the pristine monolayer. The difference in work functions between the S‐ and Se‐side surfaces (0.76 eV) was measured by Kelvin probe force microscopy, and the measurements agreed with the theoretical prediction. For the Janus MoSSe monolayer, which is terminated by the sulfur layer, the work function remains relatively uniform within the grain and is ≈4.9 eV. Conversely, the nanoscrolls (with selenium outer layer) exhibit a work function closely resembling that of graphite. Strong interlayer interactions and anisotropic optical responses of the Janus nanoscrolls were also investigated by Raman and photoluminescence (PL) spectroscopy. The Raman and PL spectra of Janus MoSSe nanoscrolls were found to exhibit a clear angle dependence with an angular periodicity of 180° and the highest values were at θ = 0° and 180°, corresponding to linear polarization along the long axis. These results experimentally confirmed the 1D structural anisotropy of the Janus nanoscrolls. Additionally, the hydrogen evolution reaction (HER) of the nanoscrolls was examined using scanning electrochemical cell microscopy (SECCM). It was found that the highly active sites for HER were localized at the edges of the nanoscrolls, likely due to the helical scrolling structure, which results in a high surface edge density.

## 6 Mechanical and Thermal Properties

The mechanical properties of individual WS_2_ nanotubes have been studied extensively and discussed before and will not be elaborated any further here. With Young's modulus of 160–170 GPa, an ultimate strength of 5–20 GPa, which is 10–100 times stronger than any polymer, and a tensile length of >10%, they present an excellent compounding additive for reinforcing various polymers. Furthermore, in the absence of strong chemical bonds between each two nanotubes, hydrogen bonds, or π‐π staking forces, these nanotubes disperse rather easily and uniformly in different polymer matrices.

More recently, the temperature dependence of the thermodynamic functions and Young's modulus of singlewall MoS_2_ and WS_2_ nanotubes were calculated as a function of temperature using optimized force field calculations.^[^ ^60^ ^]^ The Young's modulus increases with the radius of the nanotubes. Overall, while the Young's modulus displays a clear decline with rising temperatures, the reduction is not larger than 5–8 GPa from zero to 300 K. The favorable mechanical properties of multiwall WS_2_ nanotubes make them very suitable for reinforcing a variety of polymer matrices, as discussed below.

### 6.1 Electromechanical Properties

Tuning of the electrical properties of WS_2_ nanotubes via mechanical strain has been studied both theoretically and by experiment. DFT analysis of mechanically strained singlewall WS_2_ nanotubes (SWINT) was reported.^[^ ^61^ ^]^ Mechanical deformation through the tensile strain of SWINT WS_2_ nanotubes led to a linear shrinking of the bandgap and, eventually, a semiconductor‐to‐metal transition.^[^ ^61^ ^]^

**Figure 4:**
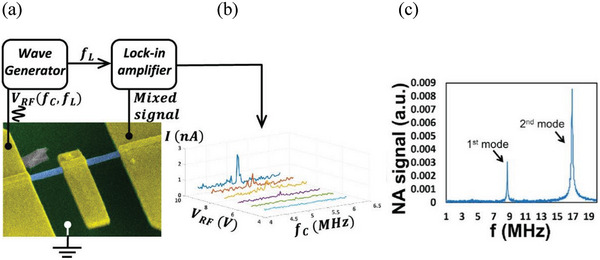
a) Self‐sensing characterization of a single WS_2_ nanotube resonator a) Illustration of the self‐sensing measurement setup (network analyzer). b) Self‐sensing frequency response under different *V*_RF_ values. c) Electrostatic characterization: Frequency response measured by the network analyzer. Adapted with permission from Ref, [62] Copyright 2022, *ACS Publications*.

More recently, all electrical torsion‐based resonator was demonstrated using multiwall WS_2_ nanotubes.^[^ ^62^ ^]^ A gold pedal was deposited asymmetrically onto a pending nanotube with two electrical contacts at its edges (**Figure** **4**). The nanotube was subjected to periodic torsion by employing frequency‐dependent electrical modulation. The electrical resistivity of the rotated nanotube was modulated, and a maximum was observed in the resonance frequency (≈5 MHz). The second harmonic of the piezoelectric torsional signal was found at ≈10 MHz. These devices could find numerous applications, such as sensors, actuators, accelerometers, and gyros.

### 6.2 Electrical Conduction and Field Emission under Mechanical Stress

**Figure 5:**
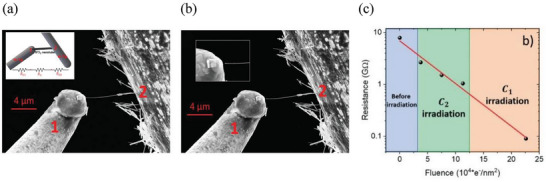
a) SEM image showing bundles and individual NTs attached to a W‐tip 2. A single, long NT, held between tips 1 and 2 by van der Waals forces, is used for electrical measurements. The inset shows the scheme of the tip 1‐WS_2_ NT‐tip 2 devices. *R*_C1_ and *R*_C2_ indicate the two resistances at the interface between the nanotube and the W‐tips, while R_3_ is the nanotube's intrinsic resistance. b) SEM image of the configuration adopted for field‐emission characterization with the single NT detached from tip 1, which is used as the anode. The inset shows a magnification of tip 1 and the nanotube‐tip region. c) Total resistance was measured as a function of the electron fluence in the two exposed areas. The red line represents an exponential fit. Adapted with permission from Ref. [63] Copyright 2020, Small, Wiley‐VCH GmbH Publications.

Di Bartolomeo et al. studied the electrical conduction, field emission under electron irradiation, and mechanical stress of individual multiwalled WS_2_ nanotubes.^[^ ^63^ ^]^ The electrical conduction of WS_2_ NTs was measured under electron beam (e‐beam) irradiation and axial strain, demonstrating a significant increase in current with e‐beam exposure. (**Figure** **5**) Carbonaceous deposits induced by e‐beam irradiation in the nanotube‐metal electrode area were employed to enhance the electrical quality of the contacts and their mechanical stability. The electron beam exposure of both contacts resulted in more than two orders of magnitude increase in the NT current.

The high aspect ratio of WS_2_ NTs (up to 100) suggested their potential for field emission applications. Indeed, it was found that an individual NT displayed a high field emission current density of 600 kA cm^−2^. Such field emission currents are higher or comparable to the maximum current density of other well‐established field emitters, such as Mo tips, carbon nanotubes, MoS_2_ nanoflowers, graphene or graphene‐like nanosheets, GaAs nanowires, etc.^[^ ^64^, ^65^, ^66^, ^67^, ^68^ ^]^ The high current density was attained with a turn‐on field of ≈100 V µm^−1^ and a field enhancement factor of ≈50.

Despite the theoretically predicted bandgap narrowing as a function of strain,^[^ ^61^ ^]^ the resistivity of multiwalled WS_2_ NTs was found to increase exponentially with the applied axial tensile stress. Namely, this study established that individual WS_2_ nanotubes are capable of withstanding over 12% strain without rupture, with resistivity exhibiting exponential growth under strain, analogous to carbon nanotubes. These findings indicate that WS_2_ nanotubes are suitable for piezoresistive strain sensor applications.

### 6.3 Thermal Conductivity

The thermal conductivity of nanostructures is a key parameter for evaluating their thermoelectric properties and exploitation of such nanostructures to various technologies. As expected, the thermal conductivity of bulk WS_2_ is highly anisotropic, and that of a single layer is 32 W mK^−1^ at room temperature.^[^ ^69^ ^]^ The (1D) thermal conductivity of a singlewall nanotube was calculated via molecular dynamics as a function of tube length, diameter, chirality, and temperature.^[^ ^69^ ^]^ For a short nanotube, the thermal conductivity is ballistic at room temperature, increasing with the NT length and having a power factor of ≈0.75. For half a micron‐long tube, the (1D) thermal conductivity is ≈80 W mk^−1^, appreciably higher than that of a 2D WS_2_ monolayer. The difference between the thermal conductivity of zigzag and armchair tubes was found to be negligible. As the temperature increases, phonon‐phonon scattering becomes predominant, and the thermal conductivity shrinks accordingly.

### 6.4 Thermoelectric Effect

Yanagi and co‐workers investigated the thermoelectric properties of a network of WS_2_ nanotubes.^[^ ^70^ ^]^ Activation of a conducting channel in the macroscopic networks of WS_2_ nanotubes in both the hole and electron regions was achieved through the application of the electrolyte‐gating technique, and an assessment of the thermoelectric properties ensued. Manipulation of the P‐ and N‐type Seebeck coefficients in the WS_2_ nanotube networks occurred by adjusting the shifts in the gate voltage potentials. The findings of this work indicated ambipolar behavior, suggesting the injection of electrons and holes through the formation of an electric double layer via electrolyte gating. It is important to emphasize that tube–tube junctions serve as scattering centers. It was suggested that enhancing the thermoelectric performance of WS_2_ nanotubes can be achieved through the construction of a more uniform nanotube network. The high thermoelectric performance of the nanotubes was found to approach that of single‐crystalline WS_2_ flakes.

## 7 Optical Properties

Nonlinear optics (NLO) explores the interaction between light and matter, with induced polarization nonlinearly dependent on an external electric field.^[^ ^71^, ^72^ ^]^ Originating in 1961 with the second harmonic generation, NLO has diverse applications, from ultrafast pulse laser generation to advanced spectroscopy, communication, and high‐resolution imaging.^[^ ^73^, ^74^ ^]^ Commercial nonlinear media currently rely on bulk crystals like beta barium borate (BBO), potassium titanyl phosphate (KTP), and lithium iodate (LiIO_3_). However, the push for miniaturization in photonic and optoelectronic devices prompts researchers to explore materials that maintain strong nonlinearity in nano‐sizes. Layered compounds, like TMDC, have revolutionized this landscape, offering unique structures and optical properties with potential applications in fundamental research and practical use.

Transition metal dichalcogenide nanotubes (TMDC NTs), synthesized over a quarter of a century ago, have primarily undisclosed optical properties. This part of the review presents the current state of knowledge on the optical characteristics of TMDC NTs, including NLO. It emphasizes their efficient performance as optical resonators with excitonic transitions contributing to emission spectra.

### 7.1 Basic Optical Properties

Electronic band structures of MoS_2_ and WS_2_ nanotubes (NTs) were investigated using density‐functional‐based tight‐binding methods.^[^ ^50^, ^75^ ^]^ The NTs, exhibiting mechanical stability, were semiconducting irrespective of the folding direction (armchair, zigzag, or chiral). The band gap increased with the NT diameter, validated experimentally through scanning tunneling microscopy (STM) and excitation Raman, with potential implications for light‐emitting devices.^[^ ^76^ ^]^ Symmetry‐based density functional theory (DFT) calculations on MoS_2_ and WS_2_ NTs revealed diameter‐dependent direct and indirect gaps influenced by curvature‐induced strain.^[^ ^77^, ^78^ ^]^ The strain effect on optical properties, including a potential transition from a direct to an indirect band gap, was explored, emphasizing the role of tube diameter.^[^ ^61^, ^79^, ^80^ ^]^ Additionally, a nonlinear saturation effect was reported, particularly pronounced in MoS_2_ NTs in aqueous suspensions.^[^ ^81^ ^]^ Nanostructures, such as WSe_2_ monolayer‐deposited arrays of pillars and MoS_2_ monolayer nanoscrolls, demonstrate intriguing optical and optoelectronic properties, including effective single‐photon emitters and light‐emitting diode effects.^[^ ^82^, ^83^, ^84^ ^]^

### 7.2 Exciton‐Polaritons

**Figure 6:**
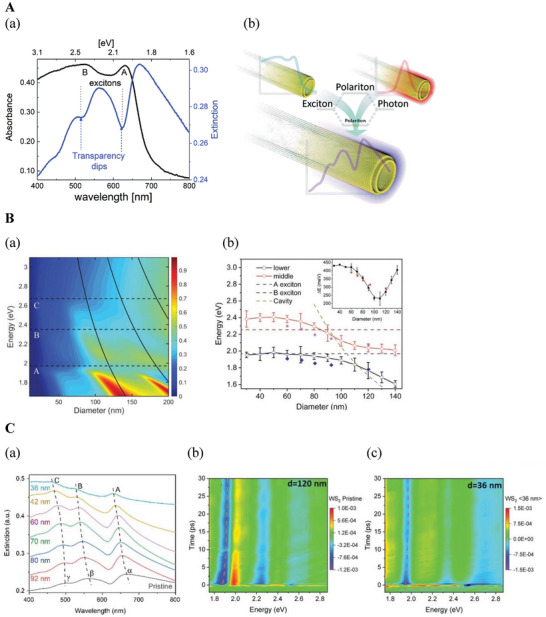
A: Steady–state absorption and extinction spectra. a) Comparison between the absolute absorbance (black) and extinction (blue) of WS_2_ nanotubes dispersed in an aqueous solution. b) Schematic illustration of the strong coupling and hybridization of optical cavity modes and excitons in the nanotubes.^[^ ^85^ ^]^ Adapted with permission from Ref. [85] Copyright 2018, Phys.Chem.Chem.Phys, RSC publications; B: a) Diameter‐dependent extinction profile of WS_2_ nanotubes, calculated from FDTD simulations (using the bulk dielectric function of WS_2_^[^ ^90^ ^]^). The dashed black lines represent the A, B, and C exciton energies. The contour is normalized to 1. The red and blue colors represent the spectrum's peaks and dips, respectively. The solid black lines indicate the dispersion of the three cavity modes within high refractive index material (*n* = 4.0). The calculations suggest that the coupling intensity diminishes for nanotubes with diameters below 60 nm. b) Simulated splitting of the two polaritonic branches in the extinction spectra as a function of nanotube diameters. The lower (black) and middle (red) polaritons are extracted from the FDTD simulations for a single nanotube diameter. The experimental points are presented as blue diamond and purple star, respectively. The A (navy) and B (brown) excitons and the cavity mode (dark yellow) are presented as dashed lines. The inset shows the splitting energy as a function of the nanotube diameter calculated from simulation (black line) and experiments (red circle). C: a) Extinction spectra of nanotubes with decreasing diameters (centrifuged sequentially in ascending speeds). Here, the α, β, and γ denote extinction peaks, and the A, B, and C denote excitons (absorption peaks) for different NTs diameters b), c) Differential transient absorbance (Δ*A*/*A*) of b) pristine NTs with an average diameter of 120 nm and c) an average diameter of 36 nm, obtained after a sequence of centrifugation steps. The color bar represents the collected signal's optical density (OD). Adapted with permission from Ref. [88] Copyright 2020, Small, Wiley‐VCH GmbH Publications.

Remarkably, WS_2_, WSe_2,_ and MoS_2_ nanotubes (NTs) sustain excitonic features and confine cavity modes in the visible–near‐infrared range, producing quasi‐particles known as exciton‐polaritons (EP).^[^ ^85^, ^86^, ^87^, ^88^ ^]^ In these studies, the extinction, absorbance, and angle‐dependent reflection spectra of aqueous dispersions and films containing NTs were investigated, confirming the EP state. The NTs exhibited cavity modes strongly coupled with the A and B excitons, resulting in additional absorbance peaks at the near IR. Notably, transparency dips were observed near the A (630 nm) and B (520) excitons absorptions. (**Figure** **6**A) The strong light‐matter interactions between the excitons and the cavity modes were confirmed through finite‐difference time‐domain (FDTD) simulations.^[^ ^85^, ^86^, ^87^, ^88^ ^]^ The simulations and the optical measurements of NTs showed that the high refraction coefficient enables them to confine cavity modes with their diameters exceeding ≈60 nm (Figure 6B). NTs of smaller diameter cannot sustain the cavity modes and display purely excitonic transitions with A and B exciton peaks.^[^ ^85^, ^86^, ^87^, ^88^ ^]^

These findings were also supported experimentally.^[^ ^87^, ^88^, ^89^ ^]^ It was demonstrated that the control over the diameter of the NTs could be achieved through a simple dispersion‐fractionation technique, allowing a transition from pure excitonic (60 nm diameter and below) to polaritonic features (diameter >80 nm). The steady–state observations were further confirmed by transient absorption experiments with the size‐fractionated nanotubes. (Figure 6C(a)) For the 36 nm diameter NTs, the recovery time for charged states was 2.1 ps, while it extends to 9.5 ps for the 120 nm diameter NTs. These timeframes are linked to the excitonic and polaritonic processes, respectively. Smaller NT shows quicker recovery, associated with excitons, while larger nanotubes have a slower recovery, indicative of polaritons‐induced relaxation. A mechanical analog of the excitons versus polaritons dynamics comes into mind by comparing the swift rebound of a rubber ball (exciton) to the gradual settling of ripples on the water surface in a pond (polariton). The tunability of the light‐matter interaction in these nanotubes opened‐up intriguing applications, including polaritonic devices, photocatalysis, and multispectral sensors.

Transient absorption measurements supported the presence of EPs in the NTs.^[^ ^85^, ^86^, ^87^, ^88^, ^91^ ^]^ It was found that the transient absorption (TA) spectrum of WS_2_ or MoS_2_ platelets differs significantly from that of NTs. Specifically, for the short delay times (≈10 fs), the light‐matter interaction is guided by excitonic absorption and resembles the TA spectrum of platelets. Meanwhile, for the long delay times (>3 ps), the process is controlled by polaritonic scattering. The coupling strength was also considered a time‐dependent entity and not a constant.^[^ ^89^ ^]^ Namely, there is a nonlinear coupling between excitonic and cavity modes and a continuous transition from weak to strong coupling limit. The TA analysis reveals that the observed shift and the longer recovery lifetimes in large‐diameter NT serve as a fingerprint of the polaritonic transient. (Figure 6C(b,c))

Additionally, Visic et al.^[^ ^91^ ^]^ developed a phenomenological coupled oscillator model with time‐dependent parameters to characterize transient extinction spectra. This model enabled an extraction of nonequilibrium electron dynamics together with bandgap renormalization. It was found that the shifts in exciton and trion resonances are induced by many‐body effects of photogenerated charge carriers and their population dynamics on the femto‐ and picosecond timescale.

### 7.3 Photoluminescence (PL) and Whispering Gallery Modes

**Figure 7:**
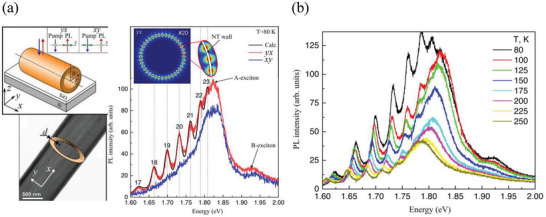
a) *Up* A sketch of a multiwall MoS_2_ nanotube on the SiO_2_/Si substrate. The studied NT has an outer radius of 1 µm and a 45‐monolayer wall determined from the fitting procedure. Blue and red lines depict the pump and PL of NT, respectively. The inset shows two different configurations of the µ‐PL experiment where green arrows indicate the light polarization. *Bottom*: A typical side‐view transmission microscopy image of a MoS_2_ NT. The cross‐section of the NT is depicted as a bright ring. The number of monolayers inside the NT wall can be estimated by dividing d/L, where L is the interlayer distance in the MoS_2_ stack (≈0.6 nm). b) Experimental spectra of the µ‐PL in the *yx* (red line) and *xy* (blue line) polarization configurations with 1 mW excitation power. The spectrum in the *yx* configuration exhibits peaks related to the optical modes. The black line shows the calculated spectrum for PL in the same configuration. The angular number of the modes is indicated above the corresponding peak. The inset represents the electric field distribution for *x*‐polarized WGM with angular number *m * =  20. c) Temperature‐dependent µ‐PL spectra in the *yx* configuration for temperatures varying from 80 to 250 K. Adapted with permission from Ref. [92] Copyright 2018, Appl. Phys. Lett., AIP Publishing.

Shubina and co‐workers presented the first evidence that a single nanotube acts as a resonator with a strong selection of modes.^[^ ^92^ ^]^ Namely, the micro‐photoluminescence (µ‐PL) spectroscopy measurements have shown emission enhancement by the strong peaks polarized along the tube axis x. (**Figure** **7**) The µ‐PL spectra of the NTs were modeled for two orthogonal experimental configurations. Perfect agreement with the experimental µ‐PL spectra has been achieved by considering the inhomogeneity of NT parameters and the frequency dependence of the refractive index. The difference between x‐polarized and y‐polarized PL spectra was explained by the distinction in angular momentum numbers of the corresponding modes in the energy region below the A‐exciton. (Figure 7a) Furthermore, they demonstrated that this selective enhancement is related to the whispering gallery modes (WGM) circulating inside the wall of the NT. The WGM peaks, strongly polarized along the tube axis, were observed in nanotubes of various diameters on silica substrates and suspended on TEM grids. It was confirmed that the observed WGMs are confined within the NT wall, situated between its inner and outer surfaces. In this work, micro‐ and nanotubes of iodine‐doped MoS_2_, ranging from 400 nm to 2 µm in diameter, exhibited radiative behavior with characteristic low‐temperature spectra. The integral PL intensity in MoS_2_ NTs was approximately an order of magnitude higher than in MoS_2_ flakes.^[^ ^93^ ^]^ At low temperatures, MoS_2_ NTs exhibit A and B excitons at 1.86 and 2.00 eV, respectively, closely matching planar atomic layers.^[^ ^94^ ^]^ The red shift of these peaks, attributed to unrelaxed strain and 3R folding polytype, is distinct from the PL in bulk crystals and persists up to room temperature in the NTs.^[^ ^78^, ^95^ ^]^ The weak emission of a phonon‐assisted indirect exciton at low temperatures is common in both MoS_2_ tubes and flakes, while the direct exciton‐related emission is prominent. As temperature rises, both tubes and flakes show two bands of direct and indirect excitons in the PL spectra, with different temperature‐dependent behaviors. The decay characteristics of the direct‐exciton PL in the NTs resemble those in monolayers rather than bulk, suggesting a more effective recombination process in the NTs.^[^ ^96^ ^]^ These findings emphasize the interconnected nature of direct and indirect exciton recombination channels in multilayered tubes and flakes.^[^ ^96^ ^]^

In an additional study, the µ‐PL of twisted micro and nanotubes with a flattened cross‐section that rotated along the tube axis was examined.^[^ ^97^, ^98^ ^]^ Despite their flattened shape, these tubes could support optical modes akin to WGM in cylindrical tubes. Through the use of µ‐PL spectroscopy for individual tubes, their distinctive feature, the splitting of WGMs, was demonstrated. This occurred when electromagnetic fields circulating on opposite tube walls could interact through a narrow gap in a highly flattened cross‐section. A model was presented, enabling the description of spectra in two types of twisted tubes: those with a constant cross‐section and those with a cross‐section varying along the tube axis (the “breathing” tubes). Both types exhibited an antiphase variation in the intensity of a pair of split modes as the cross‐section rotated. In breathing tubes, a gradual change in the splitting energy was ensured by altering the cross‐section shape along the tube axis. Twisted micro‐ and nanotubes exhibit versatile optical properties, emitting light in the 1.2–1.9 eV range and supporting optical modes with adjustable energy positions due to strong flattening of the NT cross‐section. Intrinsic strain induces cross‐section shape modulation and rotation, impacting exciton photoluminescence spectra. The split for even and odd modes in the photoluminescence spectra, sensitive to the twist angle, offer opportunities for fine‐tuning the PL through external forces, suggesting the creation of novel optomechanical devices.

### 7.4 Cathodoluminescence

**Figure 8:**
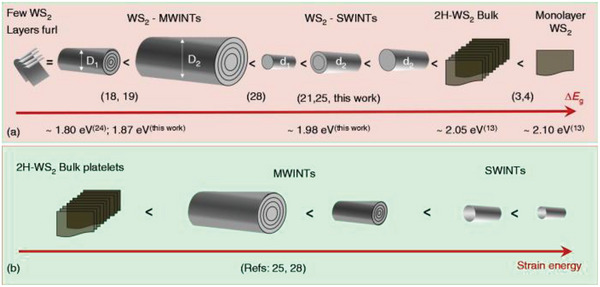
A comparative representation of the a) WS_2_ band gaps for bulk material, monolayer sheet, few folded layers, nanotubes of different diameters, and number of layers. The analysis is based on the measured CL spectra and values reported in the literature using other techniques. b) Comparative representation of the strain energy in WS_2_ bulk material and nanotubes of different diameters and number of layers as reported in the literature and the present study. (d/D – diameter of SW/MWINT, respectively). Adapted with permission from Ref. [99] Copyright 2020, App. Phys. Rev., AIP Publishing.

Zak et al. studied the optical properties of single‐wall WS_2_ nanotubes (SWINT) 3–7 nm in diameter, prepared via high‐energy plasma treatment of multiwall nanotubes (MWINTs).^[^ ^99^ ^]^ The cathodoluminescence (CL) spectra were recorded from SWINTs, MWINTs, and respective flakes (bulk). Prior studies on WS_2_/MoS_2_ nanotubes mainly focused on MWINTs, with limited exploration of SWINTs restricted to theoretical works.^[^ ^50^, ^100^ ^]^ The CL measurements revealed an intricate interplay between quantum size effects and strain in the bent triple S‐W‐S layers.^[^ ^99^ ^]^ The sharp peaks in the CL spectra were associated with direct bandgap transitions. Similar to the direct bandgap associated with the A exciton, the B exciton peak in SWINTs displays a subtle but noticeable blueshift compared to MWINTs. This experimental evidence parallels the quantum confinement effect observed in WS_2_ monolayers. The observed peak in SWINTs, attributed to the direct bandgap, exhibited a blue shift (1.98 eV) compared to MWINTs (1.87 eV), which is consistent with theoretical predictions and some experimental results for bulk WS_2_ and intercalated flakes.^[^ ^101^ ^]^ The blueshift observed in SWINTs directly reflects quantum confinement along the *c*‐axis (perpendicular to the layers) due to a reduction in the number of layers. This phenomenon is ascribed to a relatively large exciton radius, exceeding the layer thickness of 3 Å, in this direction for SWINTs. Conversely, both MWINTs and SWINTs exhibit a redshift in bandgap energies compared to the bulk material gap (2.01–2.05 eV), in agreement with previous studies, stemming from strain induced in the bent triple (S‐W‐S) layer of WS_2_ (**Figure** **8**). DFT and Time‐Dependent Density Functional Theory (TDDFT) modeling corroborated the empirical findings, highlighting an increased bandgap for monolayer tubes compared to double‐wall tubes, providing opportunities for bandgap engineering as a function of nanotube dimensions and the number of layers.

The comparative representation of the direct bandgaps for the studied nanotubes and the relevant data from the literature is depicted in Figure 8a. The compared WS_2_ nanostructures included multiwall and single‐wall nanotubes of two different diameters, a double‐wall nanotube, folded layers, a plane monolayer sheet, and bulk material. Figure 8 illustrates a qualitative representation of the strain‐energy dependence on the size and number of layers of the nanostructures.

### 7.5 Second‐Harmonic Generation

Xia et al. presented an optical second‐harmonic generation (SHG) investigation of individual multiwalled WS_2_ NTs.^[^ ^102^ ^]^ SHG microscopy, highly sensitive to crystallographic symmetry, revealed multiple structural domains and varying chirality within a single nanotube. Polarization‐resolved SHG patterns indicated distinct chiralities in different domains along the same nanotube. The study also revealed excitonic states of individual WS_2_ nanotubes through SHG excitation spectroscopy, overcoming challenges posed by the material's indirect band gap. The excitonic resonances reminiscent of those observed in TMDC monolayers were detected by analyzing the SHG excitation spectra. The measured energy of the 1s exciton in all the tubes was ≈140 meV lower than that in monolayers (2.05 eV), and the higher excitonic states exhibited features specific to each tube.

In another work, MoS_2_ nanoscrolls with various chiralities were fabricated, and their SHG performances were investigated.^[^ ^103^ ^]^ As expected from 1D material, MoS_2_ nanoscrolls exhibit reduced symmetry and a pronounced dependence of the polarization‐resolved SHG characteristics on chirality. The superposition theory of the second harmonic field of the nanoscroll walls explained the observed SHG performance. An anisotropic and chirality‐dependent SHG enhancement was revealed, which was up to two orders of magnitude larger compared to that of a MoS_2_ monolayer.

## 8 Opto‐Electro‐Mechanical Properties

### 8.1 Effect of Electric Field on the Electronic Properties

The effect of the electric field applied perpendicular to a WS_2_ (MoS_2_) nanotube axis on the electronic structure was studied via theoretical calculations.^[^ ^104^ ^]^ The bandgap of the nanotube was found to shrink linearly with the applied field, reaching the semiconductor‐to‐metal transition. The field effect was stronger by one order of magnitude for nanotubes than single‐layer WS_2_ (MoS_2_). This effect could be useful for nanoelectronic applications.

### 8.2 Transistors

In another work, small‐diameter nanotubes (≈20 nm) were evaluated for their transport characteristics. It was found that the estimated mobility values were 1.7 ± 0.5 cm^2^ V^−1^ s^−1^ for the *p*‐type and 0.26 ± 0.05 cm^2^ V^−1^ s^−1^ for the *n*‐type channels of the nanotube‐based transistor.^[^ ^105^ ^]^ An asymmetry between holes and electrons was observed, and hole mobility was found to be more than six times larger than electron mobility. Notably, the mobility values in this study were lower than those of the WS_2_ nanotube network films (discussed above).^[^ ^70^ ^]^ The authors suggested that a difference in the effective length can be one of the origins of the difference in the performances. The linear output characteristics of *p*‐ and *n*‐type channels in the devices indicated effective carrier injection. Alternative factors, like residual tungsten oxides, were considered the primary source of carrier traps, which led to the asymmetry. Moreover, the measurements were not conducted in an inert atmosphere. This aspect is crucial because potentially water absorption to the nanotube's surface may lead to charge scattering, which adversely affects the electrical properties of the nanotubes.^[^ ^106^ ^]^ Electrical transport measurements conducted with the electric double‐layer transistor (EDLT) configuration demonstrated that thin films of WS_2_ nanotubes could serve as semiconducting channels. The fabricated EDLTs exhibited distinct ambipolar operation, showcasing an on/off ratio >10^3^. This study is anticipated to expedite the utilization of nanotubes with relatively small diameters to explore the distinctive characteristics of 1D TMDC.

### 8.3 Bulk Photovoltaic Effect

**Figure 9:**
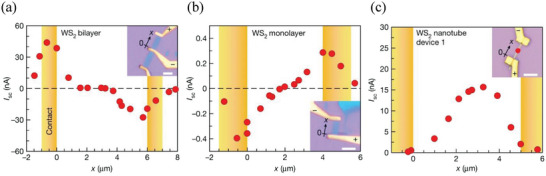
The photovoltaic response obtained with WS_2_‐based devices of different crystal symmetry. White scale bars in the optical micrographs of the devices (insets) represent 4 µm. The excitation laser wavelength was 632.8 nm in all cases. *I*_sc_ is positive when the current runs from the (+) electrode to the (–) electrode, as shown in the insets. a) The dependence of *I*_sc_ on the position of the laser spot in a WS_2_ bilayer device. *x* is the distance between the laser spot and one of the electrodes (see inset). When the laser spot illuminates the contact area, the conventional Schottky barrier photovoltaic effect and/or the photothermal effect are observed. b) The dependence of *I*_sc_ on the position of the laser spot in a WS_2_ monolayer device. Here, too, laser illumination triggers a photovoltaic response only near the contact area. c) The dependence of *I*_sc_ on the position of the laser spot in a WS_2_ nanotube device. The main response occurs when the laser spot illuminates the center of the device away from the contacts. Hence, this is a bulk photovoltaic effect. The red circle in the optical micrograph (inset) marks the position of the laser spot. Adapted with permission from Ref. [107] Copyright 2019, Nature, Springer Nature.

The bulk photovoltaic effect (BPVE) of WS_2_ nanotubes was also recently investigated. In a pivotal work, Iwasa and co‐workers investigated the BPVE in WS_2_ devices with successively lower crystal symmetry, namely centrosymmetric bilayers, non‐centrosymmetric non‐polar monolayers, and non‐centrosymmetric polar nanotubes.^[^ ^107^ ^]^ The BVPE was evaluated by measuring the short‐circuit current (*I_sc_*) under laser illumination. The authors have measured this current for WS_2_ devices with different crystal symmetry. The WS_2_ devices were fabricated with two gold contacts. Each device was systematically scanned with a laser spot 1 µm in size from one electrode to the other (**Figure** **9**). This approach aimed to differentiate the BPVE from the Schottky barrier‐induced photovoltaic effect and the photothermal effect near the contacts.^[^ ^108^, ^109^ ^]^ The authors found that in the WS_2_ bilayer and monolayer devices, a photovoltaic response is observed only when the gold contacts are illuminated by laser light, where a Schottky barrier is formed. In stark contrast, the WS_2_ nanotube device exhibited a substantial increase in photocurrent in the center of the nanotube, which was far away from the contacts. The strength of the BVPE effect in the WS_2_ NT was found to be several orders of magnitude higher than in any other material studied before. The findings of this work suggest that symmetry reduction and perhaps also a polar crystal structure were crucial for enhancing the BPVE. Nonetheless, the light‐to‐electricity conversion efficiency (<1%) was far too low for practical exploitation.

Recent theoretical work demonstrated a significant shift current (BPVE) in WS_2_ nanotubes within the infrared range.^[^ ^110^ ^]^ The essential advantage in the calculations here lies in the wall‐to‐wall charge shift, a distinctive feature of the 1D nanotube structure. A Janus‐type heteroatomic configuration was explored to enhance this inter‐wall effect. The nonlinear impact of a strong field and the nonadiabatic effect of atomic motion on the photoinduced current were evaluated by employing direct real‐time integration with time‐dependent density functional theory.

### 8.4 Flexoelectricity

The photovoltaic effect has been investigated for many decades and resulted in mature technology for solar energy exploitation. However, the efficiency of *p‐n* junction photovoltaic cells is constrained by the Schockley‐Queisser limit, which is based on thermodynamic considerations. In a search for photovoltaic devices not limited by this condition, several approaches have been discussed, like the bulk photovoltaic effect caused by the shift current and the flexoelectric effect. Lacking inversion and time‐reversal symmetry, the chiral multiwall WS_2_ nanotubes are likely to be ideally suited to exhibit such effects. Indeed, a strong bulk photovoltaic effect was demonstrated in such tubes a few years ago, as discussed above.^[^ ^107^, ^110^ ^]^ Flexoelectricity is a second‐order effect whereby strain gradient coupled in materials that lack spatial inversion symmetry and charge polarization produces (photo)electric current. This effect was investigated quantitatively in double‐wall MoS_2_ nanotubes using a theory of atomic‐bond‐relaxation and the detailed balance principle.^[^ ^111^ ^]^ The strain gradient emerged from the inner wall tube's larger curvature compared to the outer one, which is less strained. The authors calculated that as the diameter of the double‐wall NTs increases beyond 3.1 nm, the flexoelectric effect leads to a transition from type I to type II junction, whereby the photoexcited electron resides on the inner wall and the hole in the outer wall of the nanotube. The calculated flexoelectric photoconversion efficiency reaches 5.25% at a nanotube diameter of 5.2 nm. Interestingly, the authors find that the optimal photoconversion efficiency of the double wall MoS_2_ nanotubes is seven times larger than that of bilayer MoS_2_.

In another work, the generation of the flexoelectric effect of singlewall carbon and TMDC nanotubes was investigated.^[^ ^112^ ^]^ The paper discusses the size effect of the flexoelectric effect and shows that it is relatively small in bulk materials but can be large in nanoscale materials. This paper compares carbon nanotubes to TMDC nanotubes and finds that the latter exhibit a much more pronounced strain gradient due to the fact that the inner chalcogen atoms are under compression strain, while the outer chalcogen atoms are under tensile strain, which induces large strain gradient.

### 8.5 Sliding Ferroelectricity – Memory devices

The spontaneous photovoltaic effect of WS_2_ nanotubes, as detailed previously,^[^ ^70^ ^]^ covers a broad spectrum from red to blue in the visible band. This characteristic positions WS_2_ nanotubes as suitable candidates for fabricating artificial vision devices. The adjustable photovoltaic effect with a prior bias alters the rectification behavior of the WS_2_ nanotube devices, indicating changes in the electrostatic status within the nanotube. These findings suggest a flexible and programmable nature of the WS_2_ nanotube photovoltaic effect.

**Figure 10:**
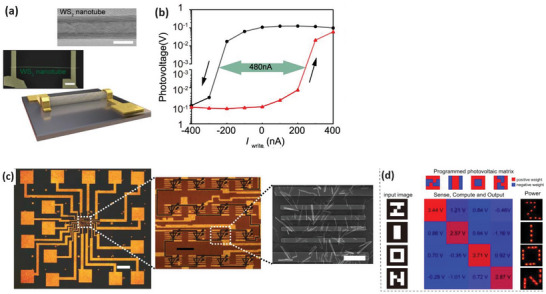
a,b) Programmability of the photovoltaic effect in WS_2_ nanotube. a) Upper: transmission electron microscopy image of a multiwall WS_2_ nanotube, scale bar: 10 nm. Middle: scanning electron microscopy image (colored) of the WS_2_ nanotube device, scale bar: 2 µm. Lower: schematic of the WS_2_ nanotube device. b) Ferroelectric‐like hysteresis loop of the photovoltaic effect through current sweep. c,d) A full‐functional artificial vision system was implanted in the PV‐RAM array. c) Optical microscopy images (left and middle) and scanning electron microscopy image of the PV‐RAM array. Scale bar left: 100 µm, middle: 20 µm, right: 5 µm. d) Demonstration of the artificial vision system. The binary weights were presented by the programmed photoresponse. The input images of “Z,” “I,” “O,” and “N” were projected onto the array by a 200 mW laser. The output voltages were collected, which indicates the probability. The output is obtained via a discharge of a capacitor, which activates a flickering LED that prints the recorded image (the word “ZION” in this case) on a paper. Adapted with permission from Ref. [113] Copyright 2022, Nature Communications, Springer Nature.

Indeed, a unique nano‐electro‐mechanical‐opto‐system was discovered within individual multiwall WS_2_ nanotubes.^[^ ^113^ ^]^ Through experimental observation and simulation, in‐plane van der Waals sliding ferroelectricity was identified, resulting from the synergy of super‐lubricity and piezoelectricity (**Figure** **10**). The hysteretic *I‐V* behavior can be attributed to a stick‐slip mechanism of the inner walls of the nanotubes, which deform and slide slightly with respect to each other under opposite bias. This mechanism induces electrical dipoles in opposite directions under opposing bias, thereby producing sliding ferroelectricity. The mesoscopic sliding ferroelectricity generates a programmable and nonvolatile photovoltaic effect in WS_2_ nanotubes, making them ideal for use as photovoltaic random‐access memory (PV‐RAM). Indeed, a device based on a four‐by‐four pixel matrix of WS_2_ NTs was fabricated, demonstrating a complete “four‐in‐one” artificial vision system (the “smart” PV‐RAM array) for detection, processing, memorization, and power supply. Both labeled supervised learning and unlabeled reinforcement learning algorithms were supported, enabling self‐driven image recognition. The recording speed of the device (≈70 ms/pixel) is compatible with other memory devices. It is important to emphasize that this important effect is pending on the multiwall structure of the WS_2_ nanotubes.

**Figure 11:**
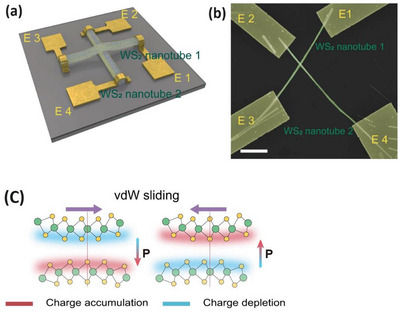
a,b) The structure and electronic property of WS_2_ nanotubes crossbar. a) Schematic diagram of the device structure. b) SEM image of the WS_2_ nanotubes crossbar. The scale bar is 3 µm. c) Mechanism and features of the 0D vdW interfacial ferroelectricity: vdW sliding along the WS_2_ 0D interface. Adapted with permission from Ref. [114] Copyright 2023, Nature Communications, Springer Nature.

In another recent work, 0D ferroelectricity was demonstrated through atomic sliding at the restrained van der Waals interface of crossed WS_2_ nanotubes.^[^ ^114^ ^]^ By stacking crossed 1D multiwall WS_2_ nanotubes, a 0D interface is produced, resulting in a spontaneous (0D) electric polarization switch via vdW sliding. The 0D (<10 nm × 10 nm × 2 nm) ferroelectric diode in this study not only presented nonvolatile resistive memory but also showcased a programmable photovoltaic effect in the visible band (**Figure** **11**). Due to the intrinsic dimensional limitation, the 0D ferroelectric diode allowed electrical operation at an ultra‐low current. By surpassing the critical size of depolarization, this work illustrated the ultimately downscaled interfacial ferroelectricity at zero‐dimension (≈100 nm^2^). It contributed to a category of devices that integrated 0D ferroelectric memory, nano‐electro‐mechanical systems, and programmable photovoltaics in one.

Notably, the primary concern lies in the low yield of the 0D sliding ferroelectric diodes, with only a small percentage (4 out of 94 devices) exhibiting rectification switch and resistive modulation. The main challenge in achieving 0D ferroelectricity is the requirement for rhombohedral stacking, which, at the current stage, is beyond reach due to the lack of chiral control in WS_2_ nanotubes. To address this, future applications necessitate the development of chirality‐controlled stacking for massive production of 0D sliding ferroelectric diodes using a network of cross‐barred multiwall WS_2_ nanotubes.

### 8.6 Superconducting Diode and Paraconductivity

Asymmetric (nonreciprocal) *I–V* curves in semiconductor junctions make the heart of the electronic industry and are produced by the inherent asymmetry of the *p‐n* junction (and Schottky diodes). The *I–V* curve of superconducting materials is usually symmetric, and the current going from left to right is indistinguishable from the one in the opposite direction. However, much interest has recently been devoted to superconductor diodes wherein the currents in the two directions are asymmetric and hence could be the source of a new electronic technology. For that to occur, the (spatial) inversion and time reversal symmetry must be revoked. Chiral nanotubes are an ideal platform for such a phenomenon. Their chiral nature violates the spatial inversion symmetry. Applying a magnetic field in an axial direction of the nanotubes breaks the time‐reversal symmetry, producing the so‐called magneto‐chiral anisotropy or superconducting diode effect.^[^ ^115^, ^116^ ^]^ A related effect, i.e., the nonreciprocal paraconductivity, is also discussed here.^[^ ^115^ ^]^ Paraconductivity is the current produced by the electrons that are partially paired (Cooper pairs), but they do not form a coherent superconducting condensate. This effect has been known for many years and is observed slightly above the critical temperature (Tc). Using the same formalism (Ref. [115]), the nonreciprocity of the paraconductivity is calculated for the chiral tubes.

## 9 Applications

### 9.1 Polymer Nanocomposites

The antiballistic properties of polymer nanocomposites are potentially important in different civil and military applications. For example, using higher impact‐resistant nanocomposites for transportation could improve safety, save lives, and reduce fuel consumption and the carbon footprint of the vehicle. To this end, small amounts of WS_2_ nanotubes were compounded with polymethyl‐methacrylates (PMMA), which exhibited up to 31% improvement in toughness in the Split‐Hopkinson pressure bar test.^[^ ^117^ ^]^ Poly L‐lactic acid (PLLA) is a biocompatible and biodegradable polymer that is extensively used in medical technology. In particular, the application of PLLA in bioresorbable cardiovascular catheters (BVS) to treat coronary heart diseases is a highly promising technology. However, the low mechanical strength of the polymer does not afford BVS of proper dimensions, and therefore, their medical applications were banned after a short period of usage. In an attempt to address this issue as well as increase the radiopacity of the device, PLLA‐WS_2_ NTs nanocomposites were prepared and studied.^[^ ^118^ ^]^ Extrusion of this nanocomposite and its solidification were studied *in‐operando* using wide‐angle X‐ray scattering (WAXS) and small‐angle X‐ray scattering (SAXS) in the synchrotron. The nanotubes were found to disperse excellently in the polymer blend, orient axially along the filament, and induce nucleation of the polymer crystallites, thereby mechanically reinforcing the nanocomposite string. Further studies of this system showed that the nanotubes are not cytotoxic and increase radiopacity.^[^ ^119^ ^]^ In another study,^[^ ^120^ ^]^ small amounts of WS_2_ NTs were melted and extruded with polylactic acid (PLA) polymer. Subsequently, the “green” filaments were used as feedstock for the 3D printing films. A factor of three enhancement in the fracture toughness was observed in the printed PLA films upon adding 0.5 wt.% of WS_2_ nanotubes. These studies offer a myriad of applications of WS_2_ in various medical technologies.

### 9.2 Li‐Ion Batteries

The search for clean energy sources has invigorated research into rechargeable Li‐ion batteries in recent decades. TMDCs were investigated as cathode materials in Li‐intercalation batteries for over 50 years. With a theoretical capacity of 432 mA h g^−1^ coupled with chemical and structural robustness, nanoparticles of WS_2_ are a promising material for Li intercalation batteries.^[^ ^121^ ^]^ Here, fullerene‐like WS_2_ nanoparticles 15 nm in diameter were anchored to graphene by first ball milling WO_3_ nanoparticles with graphene and subsequent sulfidation of the product at elevated temperatures. To improve the structural stability and electrochemical cycling of the hybrid material, the WS_2_ NPs were coated with a thin amorphous carbon film by adding glucose to the ball‐milled precursors. The electrode was cycled at a high charge‐discharge current of 1000 mA g^−1^, showing a remarkable capacity of 371.9 mA h g^−1^ and 62% retention of the capacity after 500 cycles with no further fading of the capacity.

In another work, WS_2_ nanotubes were impregnated into polymer‐silicon oxycarbide (SiOC) fibers via electrospinning followed by cross‐linking at 160 °C and pyrolysis at 800 °C.^[^ ^122^ ^]^ The Li‐ion cell exhibited a high initial capacity of 454 mAh g^−1^ with moderate capacity retention. Further research is being undertaken to understand the capacity fading mechanism and to use sodium ions for the intercalation battery.

### 9.3 Fuel Cell (Membranes)

Fuel cell technology is considered the next generation of clean energy sources. Nonetheless, their commercial exploitation is hindered by both technological hurdles and cost considerations. Highly conductive proton exchange membranes, which permit proton transport in one direction and block transport of larger ions across the two half cells, are a critical component of high‐performance fuel cells and other applications. In a recent work, membranes with straight proton channels were manufactured by decorating WS_2_ nanotubes with magnetite (Fe_3_O_4_) nanoparticles.^[^ ^123^ ^]^ Application of a magnetic field on a slurry of Nafion (sulfonate‐group terminated perfluoro polymer) and magnetite‐loaded WS_2_ nanotubes led to aligning the nanotubes across the Nafion membrane. The Nafion with magnetite‐loaded WS_2_ nanotubes composite exhibited 69% higher proton conductivity and 51% higher power compared to the pure Nafion membrane. Molecular dynamics modeling suggested that the enhanced proton conduction occurs at the interface between the sulfate groups of the Nafion, which are anchored to the nanotube surface.

### 9.4 Liquid Crystal Display Devices

Liquid crystals (LC) play a major role in display devices like computer screens and television sets. Being long molecules with large electrical dipoles, they form quasi‐ordered mesophases that exhibit large optical anisotropy (birefringence). Therefore, the transparency of an LC pixel can be modulated via high‐speed switching of the electric field. The switching speed is determined by various parameters, like the anisotropy in the dielectric permittivity, viscosity of the mesophase, amplitude of the electric field, etc. Adding WS_2_ nanotubes to LC mesophases was shown to increase the switching speed of the device.^[^ ^124^, ^125^ ^]^ The investigation suggested that the LC molecules are oriented along the nanotube axis, offering a fast reorientation mechanism for the mesophase's domains. Obviously, beyond a certain concentration (say 0.3 wt.%), the black color of the nanotubes becomes prohibitive, blocking the transmission of light through the LC film.

### 9.5 Water Splitting

Generally speaking, multiwall WS_2_ nanotubes with diameters between 40–120 nm are not expected to reveal high catalytic reactivity since they expose the inert basal plane to the solution, and the bending strain is not particularly large. However, this picture may change substantially for small‐diameter nanotubes owing to the large strain effect and the highly distorted chemical bonds, which may further their surface reactivity. One way to promote the catalytic reactivity of such nanotubes in chemical and electrochemical reactions is by depositing tiny metal nanoparticles on the nanotube's surface or doping it with, e.g., transition metal atoms.

Arguably, one of the most explored catalytic reactions is water splitting, particularly the photocatalytic generation of hydrogen (and oxygen), considered the cleanest fuel. Semiconductor electrodes must comply with several stringent requirements to convert sunlight and split water molecules efficiently. The first condition that must be fulfilled is that the energy gap of the semiconductor should be larger than the free energy of the water‐splitting reaction, i.e., >1.23 eV. Furthermore, the conduction band‐edge must be higher in energy (more negative potential) than the hydrogen level (redox potential), and the valence band‐edge must be positioned below the oxygen level. These requisites are discussed through computational work in,^[^ ^126^ ^]^ where single‐ to triple‐wall WS_2_ nanotubes of different chiralities and diameters were considered for the water‐splitting reaction.

**Figure 12:**
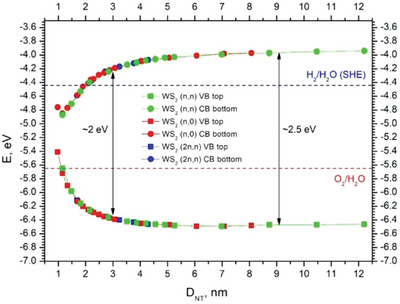
Dependence of valence band and conduction band edges on the WS_2_ NT diameter for three different configurations: achiral (*n*, *n*) and (*n*, 0), as well as chiral (2*n*, *n*). Energy is referenced to a vacuum level. Adapted with permission from Ref. [126] Copyright 2019, ACS Omega., ACS Publications.

**Figure** **12** shows the results of the calculations for singlewall zigzag (n,0), armchair (n,n), and chiral (2n,n) nanotubes. Here, a nanotube with a diameter >2 nm can potentially be useful for the water‐splitting reaction. Notably, the bandgap of the nanotubes increases with increasing diameter of the tubes. While the valence band of the nanotubes increases, it goes down, crossing the oxygen level at a diameter of ≈1 nm. The crossover point of the conduction band and the hydrogen level is ≈2 nm. Interestingly, the chirality of the nanotube does not seem to play any role in this process. This actually means that the nanotubes can exploit photons covering most of the visible and infrared spectrum of sunlight to drive this reaction. Nanotubes of such small diameter suffer considerable strain, and their chemical bonds are highly distorted, which makes them potentially reactive with respect to catalytic reactions.

### 9.6 Photocatalysis

Recently, WS_2_ nanotubes and fullerene‐like nanoparticles were found to be effective photocatalysts in the photocuring of acrylate‐based polymer films.^[^ ^127^ ^]^ Adding the nanotubes/IF nanoparticles into a polymer blend containing a commercial photocuring agent (Irgacure 819) led to a substantial acceleration of the photocuring process. Using electron paramagnetic resonance (EPR), the photocuring mechanism of the inorganic nanoparticles was found to be entirely different from that of the commercial photocuring agent. Moreover, mechanical tests demonstrated that, unlike the commercial photocuring agent, which has no influence on the film properties, the IF/INT substantially improved the fracture toughness of the photocured film. This development suggests a plethora of applications for nanoparticles in different technologies, including 3D printing.

In another study, WS_2_ nanotubes (NTs) played a pivotal role in improving the stability and photocatalytic performance of halide perovskite nanocrystals (HP‐NCs).^[^ ^128^, ^129^ ^]^ Steric stabilization was attained by combining HP‐NCs with WS_2_ NTs, even with a modest amount of the NTs (≈11 wt.%). The nanocomposite demonstrated increased absorbance cross‐sections and exhibited ultrafast charge transfer between HP‐NCs and the WS_2_ NTs. Moreover, the HP/WS_2_ NTs nanocomposites emerged as superior photocatalysts for dye degradation in polar solvents.

Finally, it is believed that some of the recent optoelectronic devices discussed in this review, and in particular the torsion resonator,^[^ ^62^ ^]^ the optical memory device,^[^ ^113^ ^]^ and the 0D ferroelectric node,^[^ ^114^ ^]^ are rather promising technologies that call for further research and development efforts.

## 10 Conclusions

Although TMDC nanotubes were discovered shortly after carbon nanotubes, they received much less attention, likely due to the fact that they cannot be easily produced as singlewall tubes with well‐defined chiral angles. The difficulty in producing them as singlewall nanotubes with well‐tuned chirality are not accidental and stems from physical reasons. Past DFT calculations have confirmed that TMDC nanotubes are more stable in the multiwall structure. This makes them possibly less popular materials for investigation compared to single‐layer TMDCs and SWCNT. Nonetheless, recent progress in their production as a single phase with high crystalline order and the general interest in 2D materials brought them back into the research limelight. Recent progress in the fabrication of TMDCs nanoscrolls with well‐controlled chirality and number of walls could be a good starting point for studying quasi‐1D nanostructures for a variety of applications.^[^ ^130^ ^]^ Several other frontiers in the synthesis and research of TMDC nanotubes are: 1. Synthesis of cm‐long multiwall WS_2_ nanotubes in substantial amounts, which could find applications as filtration membranes and for fabrication of integrated memory devices.^[^ ^114^ ^]^ Synthesis of the elusive Janus nanotubes, like Se‐Mo‐S and others. The calculations pointed out above (see Sec. 4) suggest numerous intriguing properties for such 1D nanostructures, e.g., in the generation of non‐linear optical signals. Core‐shell nanotubes present another promising route for research. So far, not much has been achieved in this respect other than the synthesis of limited amounts of a variety of core‐shell nanotubular structures. Core‐shell nanotubes could display interesting (1D) optical and electrical properties akin to heterojunction in flat 2D materials, like type II excitons. The multiwall structure of such nanotubes was found to be advantageous for numerous studies in the field of energy conversion and storage, polymer nanocomposites, and, more recently, in unique optoelectronic devices. These studies offer myriad potential applications for TMDC nanotubes in a variety of technologies, including bio‐medical technologies, which must be worked out in greater detail in future research. Given the good electro‐optical properties of TMDC nanotubes, one would expect to have much more work done on the photocatalytic applications of such nanotubes. It must be born in mind, however, that fullerene‐like nanoparticles of WS_2_ have been commercialized as superior solid lubricants, and joint academia‐industry efforts are underway to commercialize polymer nanocomposites reinforced with WS_2_ nanotubes in a variety of technologies. All in all, therefore, there is a huge untapped potential for these 1D nanotubular structures for further research and applications.

## Conflict of Interest

The authors declare no conflict of interest.
